# Dysphagia in Parkinson´s disease. A 5-year follow-up study

**DOI:** 10.1007/s10072-025-08027-8

**Published:** 2025-02-19

**Authors:** Diego Santos-García, Teresa de Deus Fonticoba, Silvia Jesús, Marina Cosgaya, Juan García Caldentey, Nuria Caballol, Ines Legarda, Jorge Hernández Vara, Iria Cabo, Lydia López Manzanares, Isabel González Aramburu, Maria A. Ávila Rivera, Víctor Gómez Mayordomo, Víctor Nogueira, Julio Dotor García-Soto, Carmen Borrué, Berta Solano Vila, María Álvarez Sauco, Lydia Vela, Sonia Escalante, Esther Cubo, Zebenzui Mendoza, Isabel Pareés, Pilar Sánchez Alonso, Maria G. Alonso Losada, Nuria López Ariztegui, Itziar Gastón, Jaime Kulisevsky, Manuel Seijo, Caridad Valero, Ruben Alonso Redondo, Carlos Ordás, Manuel Menéndez-González, Darrian McAfee, Pablo Martinez-Martin, Pablo Mir, COPPADIS Study Group, A. D. Adarmes, M. Almeria, M. G. Alonso Losada, A. Alonso Cánovas, F. Alonso Frech, R. Alonso Redondo, I. Álvarez, M. Álvarez Sauco, A. Aneiros Díaz, S. Arnáiz, S. Arribas, A. Ascunce Vidondo, M. Aguilar, M. A. Ávila, N. Bernardo Lambrich, H. Bejr-Kasem, M. Blázquez Estrada, M. Botí, C. Borrue, M. T. Buongiorno, C. Cabello González, I. Cabo López, N. Caballol, A. Cámara Lorenzo, H. Canfield Medina, E. Carabajal Pendón, F. Carrillo, F. J. Carrillo Padilla, E. Casas, M. J. Catalán, P. Clavero, A. Cortina Fernández, M. Cosgaya, A. Cots Foraster, A. Crespo Cuevas, E. Cubo, T. de Deus Fonticoba, O. de Fábregues-Boixar, M. Díez-Fairen, J. Dotor García-Soto, E. Erro, S. Escalante, E. Estelrich Peyret, N. Fernández Guillán, P. Gámez, M. Gallego, J. García Caldentey, C. García Campos, C. García Díez, J. M. García Moreno, I. Gastón, M. P. Gómez Garre, V. Gómez Mayordomo, J. González Aloy, I. González-Aramburu, J. González Ardura, B. González García, M. J. González Palmás, G. R. González Toledo, A. Golpe Díaz, M. Grau Solá, G. Guardia, J. Hernández Vara, A. Horta-Barba, D. Idoate Calderón, J. Infante, S. Jesús, J. Kulisevsky, M. Kurtis, C. Labandeira, M. A. Labrador, F. Lacruz, M. Lage Castro, S. Lastres Gómez, I. Legarda, N. López Ariztegui, L. M. López Díaz, D. López Domínguez, L. López Manzanares, B. López Seoane, S. del Lucas Pozo, Y. Macías, M. Mata, G. Martí Andres, M. J. Martí, J. C. Martínez Castrillo, P. Martinez-Martin, D. McAfee, M. T. Meitín, Z. Mendoza Plasencia, M. Menéndez González, C. Méndez del Barrio, P. Mir, J. Miranda Santiago, M. I. Morales Casado, A. Moreno Diéguez, I. Muro García, V. Nogueira, A. Novo Amado, S. Novo Ponte, C. Ordás, J. Pagonabarraga, I. Pareés, B. Pascual-Sedano, P. Pastor, A. Pérez Fuertes, R. Pérez Noguera, A. Planas-Ballvé, L. Planellas, M. A. Prats, C. Prieto Jurczynska, V. Puente, M. Pueyo Morlans, A. Puig Daví, N. Redondo Rafales, L. Rodríguez Méndez, A. B. Rodríguez Pérez, F. Roldán, M. Ruíz De Arcos, J. Ruíz Martínez, P. Sánchez Alonso, M. Sánchez-Carpintero, G. Sánchez Díez, A. Sánchez Rodríguez, P. Santacruz, D. Santos García, J. C. Segundo Rodríguez, M. Seijo, M. Sierra Peña, B. Solano Vila, E. Suárez Castro, J. P. Tartari, C. Valero, L. Vargas, L. Vela, C. Villanueva, B. Vives

**Affiliations:** 1 Department of Neurology, Hospital Universitario de A Coruña (HUAC), Complejo Hospitalario Universitario de A Coruña (CHUAC), C/ As Xubias 84, 15006 A Coruña, Spain; 2 Grupo de Investigación en Enfermedad de Parkinson y Otros Trastornos del Movimiento, INIBIC (Instituto de Investigación Biomédica de A Coruña), A Coruña, Spain; 3 Hospital San Rafael, A Coruña, Spain; 4 Fundación Degen, A Coruña, Spain; 5 CHUF, Complejo Hospitalario Universitario de Ferrol, A Coruña, Spain; 6 Unidad de Trastornos del Movimiento, Servicio de Neurología y Neurofisiología Clínica, Instituto de Biomedicina de Sevilla, Hospital Universitario Virgen del Rocío/CSIC/Universidad de Sevilla, Seville, Spain; 7 CIBERNED (Centro de Investigación Biomédica en Red Enfermedades Neurodegenerativas), Madrid, Spain; 8 Hospital Clínic de Barcelona, Barcelona, Spain; 9 Centro Neurológico Oms 42, Palma, Spain; 10 Consorci Sanitari Integral, Hospital Moisés Broggi, Sant Joan Despí, Barcelona, Spain; 11 Hospital Universitario Son Espases, Palma, Spain; 12 Hospital Universitario Vall d´Hebron, Barcelona, Spain; 13 Complejo Hospitalario Universitario de Pontevedra (CHOP), Pontevedra, Spain; 14 Hospital Universitario La Princesa, Madrid, Spain; 15 Hospital Universitario Marqués de Valdecilla - IDIVAL, Santander, Spain; 16 Consorci Sanitari Integral, Hospital General de L´Hospitalet, L´Hospitalet de Llobregat, Barcelona, Spain; 17 Neurology Department, Institute of Neuroscience, Vithas Madrid La Milagrosa University Hospital, Vithas Hospital Group, Madrid, Spain; 18 Hospital Universitario Lucus Augusti, Lugo, Spain; 19 Hospital Universitario Virgen Macarena, Seville, Spain; 20 Hospital Infanta Sofía, Madrid, Spain; 21 Institut d‘Assistència Sanitària (IAS) - Institut Català de La Salut, Girona, Spain; 22 Hospital General Universitario de Elche, Elche, Spain; 23 Fundación Hospital de Alcorcón, Madrid, Spain; 24 Hospital de Tortosa Verge de La Cinta (HTVC), Tortosa, Tarragona, Spain; 25 Complejo Asistencial Universitario de Burgos, Burgos, Spain; 26 Hospital Universitario de Canarias, San Cristóbal de La Laguna, Santa Cruz de Tenerife, Spain; 27 Hospital Universitario Ramón y Cajal, IRYCIS, Madrid, Spain; 28 Hospital Universitario Puerta de Hierro, Madrid, Spain; 29 Hospital Álvaro Cunqueiro, Complejo Hospitalario Universitario de Vigo (CHUVI), Vigo, Spain; 30 Complejo Hospitalario de Toledo, Toledo, Spain; 31 Complejo Hospitalario de Navarra, Pamplona, Spain; 32 Hospital de Sant Pau, Barcelona, Spain; 33 Hospital Arnau de Vilanova, Valencia, Spain; 34 Hospital Rey Juan Carlos, Madrid, Spain; 35 Hospital Universitario Central de Asturias, Oviedo, Spain; 36 University of Maryland School of Medicine, Baltimore, MD USA; 37 Departamento de Medicina, Facultad de Medicina, Universidad de Sevilla, Seville, Spain

**Keywords:** Cohort, Early, Dysphagia, Impulse control disorder, Non-motor symptoms, Parkinson's disease

## Abstract

**Background and objective:**

Dysphagia at time of diagnosis suggests atypical parkinsonism instead Parkinson´s disease (PD). Our aim was to analyze the frequency of dysphagia in patients with early PD comparing with a control group and to identify related factors.

**Patients and methods:**

Patients with early PD (≤ 2 years from symptoms onset) who were recruited from January/2016 to November/2017 (baseline visit; V0) and evaluated annually for 5 years from the Spanish cohort COPPADIS were included in this prospective study. Controls were assessed at baseline and at 2-, 4-, and 5-year follow-up. Dysphagia was defined as a score ≥ 1 in the item 20 of the Non-Motor Symptoms Scale (NMSS).

**Results:**

Dysphagia was more frequent at baseline in PD patients (19.6% [36/184]; 62.3 ± 8.3 years old; 56.8% males) than in controls (5.3% [11/206]; 60.9 ± 8.3 years old; 50% males) (*p* < 0.0001) and in all visits as well (*p* < 0.0001). A worse quality of sleep (Parkinson´s Disease Sleep Scale; OR = 0.974; *p* = 0.005), a greater impulse-control behavior (ICB) (Questionnaire for Impulsive-Compulsive Disorders in Parkinson's Disease-Rating Scale; OR = 1.066; *p* = 0.014), and non-motor symptoms burden (Non-Motor Symptoms Scale; OR = 1.016; *p* = 0.021) were independent factors associated with dysphagia at baseline. In those subjects with dysphagia, no differences were observed between patients and controls in the mean NMSS-item 20 overtime, and it didn´t change throughout the follow-up.

**Conclusion:**

Dysphagia was frequent in early PD patients compared to controls. However, it was minor and did not progress over time. Sleep, ICB, and non-motor symptoms burden were related to dysphagia.

**Supplementary Information:**

The online version contains supplementary material available at 10.1007/s10072-025-08027-8.

## Introduction

Dysphagia is highly prevalent in Parkinson disease (PD) but is not typically identified nor treated until later in the disease process. In fact, severe dysphagia within the first 5 years is a known red flag for diagnosis of PD [1]. Dysphagia in PD varies significantly when it is based on subjective than on objective measurements [2]. A recent meta-analysis found a pooled prevalence rate of dysphagia in PD of 36.9% (95% CI: 30.7–43.6%), whereas instrumental examination showed a higher prevalence (57.3%, 95% CI: 44.3–69.1%) [3]. Dysphagia is associated with older age, male sex, lower body mass index, longer disease duration, higher Hoehn and Yahr (H&Y) stage and levodopa equivalent daily dose (LEDD), PIGD (Postural Instability Gait Difficulties) subtype, severe motor symptoms, cognitive impairment, drooling, higher levels of depression, and lower quality of life (QoL) [3–5]. Furthermore, dysphagia reduces quality of life (QoL), complicates medication intake, predicts a worse outcome in late-stage PD [6] and plays a role in weight loss and recurrent of airway infections [7]. However, dysphagia can be present at early phases of PD as well and often is underestimated. In fact, dysphagia has not been well studied in early PD [8]. In a recent meta-analysis about dysphagia prevalence and associated factors in PD involving 58 studies [3], the mean disease duration was 7.4 (range from 0.5 to 17.8), and only 2 studies had a mean disease duration of 2 years or less [9, 10]. The prevalence of dysphagia in a cohort of early drug-naïve PD patients from the PPMI database was 12.3% (49/398) with a mean disease duration of 0.5 ± 0.5 years [9]. Dysphagia occurred in 20.1% (349/1738) of the patients from the United Kingdom Tracking Parkinson's Study with recent onset symptoms (mean disease duration 1.3 ± 0.9 years) [10]. However, both studies were cross-sectional and without a control group. Thus, more information about the role of dysphagia in early PD is needed.

The aim of the present study was to analyze the frequency of dysphagia in patients with early PD, and it to compare with a control group. Specifically, we identified the prevalence of dysphagia, the burden of the symptom, and its change over a 5-year span in patients and controls. Moreover, we identified factors related to dysphagia and analyzed the correlation of dysphagia with drooling, hypomimia, and speech problems.

## Material and methods

Patients with early PD (≤ 2 years from symptoms onset) who were recruited from January/2016 to November/2017 (baseline visit; V0) and evaluated annually for 5 years from the Spanish cohort COPPADIS [11] were included in this longitudinal prospective study. This is a multi-center, observational, prospective, 5-year follow-up study designed to analyze disease progression in a Spanish population of PD patients. Methodology about COPPADIS-2015 study can be consulted in https://bmcneurol.biomedcentral.com/articles/10.1186/s12883-016-0548-9 [12]. All patients included at baseline were diagnosed according to UK PD Brain Bank criteria [13]. Exclusion criteria were: atypical parkinsonism; Mini Mental State Examination [MMSE] < 26; age < 18 or > 75 years; inability to read or understand the questionnaires; to receive any advanced therapy (continuous infusion of levodopa or apomorphine; and/or with deep brain stimulation at baseline); and the presence of comorbidities, sequelae, or any other disorder that could interfere with the assessment. Control subjects from the COPPADIS cohort assessed at baseline and at 2-, 4-, and 5-year follow-up were also included.

At each visit, to have dysphagia was defined as a non-zero score (from 1 to 12) in the item 20 (“Does the patient having difficulty swallowing?”) of the Non-Motor Symptoms Scale (NMSS) [14]. A score ≥ 6 was considered as relevant dysphagia burden (from often x severe or frequent x moderate [6 points] to always x severe [12 points]). The same method was used to define drooling, according to the NMSS-item 19 (Does the patient dribble saliva during the day?). Regarding speech problems, PD patients were classified in 5 groups according to the item-18 of the Unified Parkinson´s Disease Rating Scale – part III (UPDRS-III): 0 = Normal; 1 = Slight loss of expression, diction and/or volume; 2 = Monotone, slurred but understandable; moderately impaired; 3 = Marked impairment, difficult to understand; 4 = Unintelligible [15]. The UPDRS-III was also used to define facial expression (item-19): 0 = Normal; 1 = Minimal hypomimia, could be normal "Poker Face"; 2 = Slight but definitely abnormal diminution of facial expression; 3 = Moderate hypomimia; lips parted some of the time; 4 = Masked or fixed facies with severe or complete loss of facial expression; lips parted 1/4 inch or more.

Information on sociodemographic aspects, factors related to PD, and treatment was collected. The H&Y, UPDRS-III and part IV (UPDRS-IV), NMSS and ADLS (Schwab & England Activities of Daily Living Scale) were applied annually in PD patients. Other data about motor status, non-motor symptoms (NMS) and QoL were assessed at V0, V2, V4, and V5 using different validated scales: Freezing of Gait Questionnaire (FOGQ); Beck Depression Inventory-II (BDI-II); Parkinson's Disease Sleep Scale (PDSS); Neuropsychiatric Inventory (NPI); Questionnaire for Impulsive-Compulsive Disorders in Parkinson's Disease-Rating Scale (QUIP-RS); Visual Analog Scale-Pain (VAS-Pain); Visual Analog Fatigue Scale (VAFS]); the 39-item Parkinson's disease Questionnaire (PDQ-39); the EUROHIS-QOL 8-item index (EUROHIS-QOL8) [12]. In patients with motor fluctuations, the motor assessment was made during the OFF state (without medication in the last 12 h) and during the ON state. The assessment was only performed without medication in patients without motor fluctuations. Motor phenotype, motor fluctuations, FOG, falls, and major depression were defined in the COPPADIS protocol according to the literature and scales used [12]. Moreover, impulse control behaviors (ICBs) were considered, both impulse control disorders (ICDs) (pathological gambling, compulsive shopping, hypersexuality, and compulsive eating behavior) and compulsive behaviors (CBs) (punding, hobbyism, and dysregulation dopaminergic syndrome). We applied previously published cutoff points of the QUIP-RS: gambling ≥ 6, buying ≥ 8, sex ≥ 8, eating ≥ 7, hobbyism-punding ≥ 7 [16]. For dopaminergic dysregulation syndrome (DDS), we accounted for the researcher´s criteria, since an established cutoff does not exist [17]. Patients suffering from at least one ICD and/or CB were considered as patients presenting ICB. The same evaluation as for the patients, except for the motor assessment, was performed in control subjects at the same visits (V0; V2; V4; V5). LEED was calculated based on the literature [18].

### Statistical analysis

Data were processed using SPSS 20.0 for Windows. Different variables were expressed as quantitative and/or qualitative variables. Distribution of variables was verified by one-sample Kolmogorov–Smirnov test. All subjects from the COPPADIS cohort who met defined criteria were selected for comparisons at the baseline visit. However, it was mandatory that all selected participants must have been evaluated at all visits to compare the changes in the follow-up: V0; V1 (at 1-year); V2 (at 2-year); V3 (at 2-year); V4 (at 4-year); V5 (at 5-year).

For comparisons between subjects (patients vs controls) and from a different group (with vs without dysphagia) at each visit (V0; V2; V4; V5), the Student's t-test, Mann–Whitney U, Chi square, or Fisher test were applied. Binary regression models were used to determine independent factors associated with dysphagia (dysphagia as dependent variable). Variables with univariate associations with p-values < 0.20 were included in a multivariable model, and a backwards selection process was used to remove variables individually until all remaining variables were significant at the 0.10 level except age, gender, disease duration and LEDD, added as covariates. Spearman’s or Pearson’s correlation coefficient, as appropriate, were used for analyzing the relationship between dysphagia and drooling, speech problems, and hypomimia. Correlations were considered weak for coefficient values ≤ 0.29, moderate for values between 0.30 and 0.59, and strong for values ≥ 0.60. General linear model (GLM) repeated measures were used to test for changes in the mean NMSS-item 20 score in PD patients and in controls. In the models, age, gender, disease duration, and LEED at each visit were included as covariates for PD patients and age and gender for controls. The Bonferroni method was used as a post-hoc test after ANOVA. Cohen´s d formula was applied for measuring the effect size. It was considered: < 0.2 – Negligible; 0.2 – 0.49 – Small; 0.50 – 0.79 – Moderate; ≥ 0.80 – Large. The value of p was considered significant when it was < 0.05.

### Standard protocol approvals, registrations, and patient consents

For this study, we received approval from the *Comité de Ética de la Investigación Clínica de Galicia* from Spain (2014/534; 02/DEC/2014). Written informed consents from all participants (patients and controls) in this study were obtained.

## Results

A total of 184 early PD patients (62.3 ± 8.3 years old; 56.8% males) and 206 controls (60.9 ± 8.3 years old; 50% males) were included. Mean disease duration was 1.3 ± 0.7 years. At baseline, dysphagia was significantly more frequent in PD patients than controls (19.6% vs 5.3%; *p* < 0.0001) and mean score of the NMSS-item 20 was significantly higher in PD patients (0.4 ± 1 vs 0.1 ± 0.8; *p* < 0.0001). However, only one patient had relevant dysphagia (NMSS-item 20 ≥ 6) and no differences were detected in the NMSS-item 20 score in patients compared to controls when only subjects with dysphagia were included (2.1 ± 1.3 vs 2.4 ± 2.4; *p* = 0.685).

**Fig. 1:**
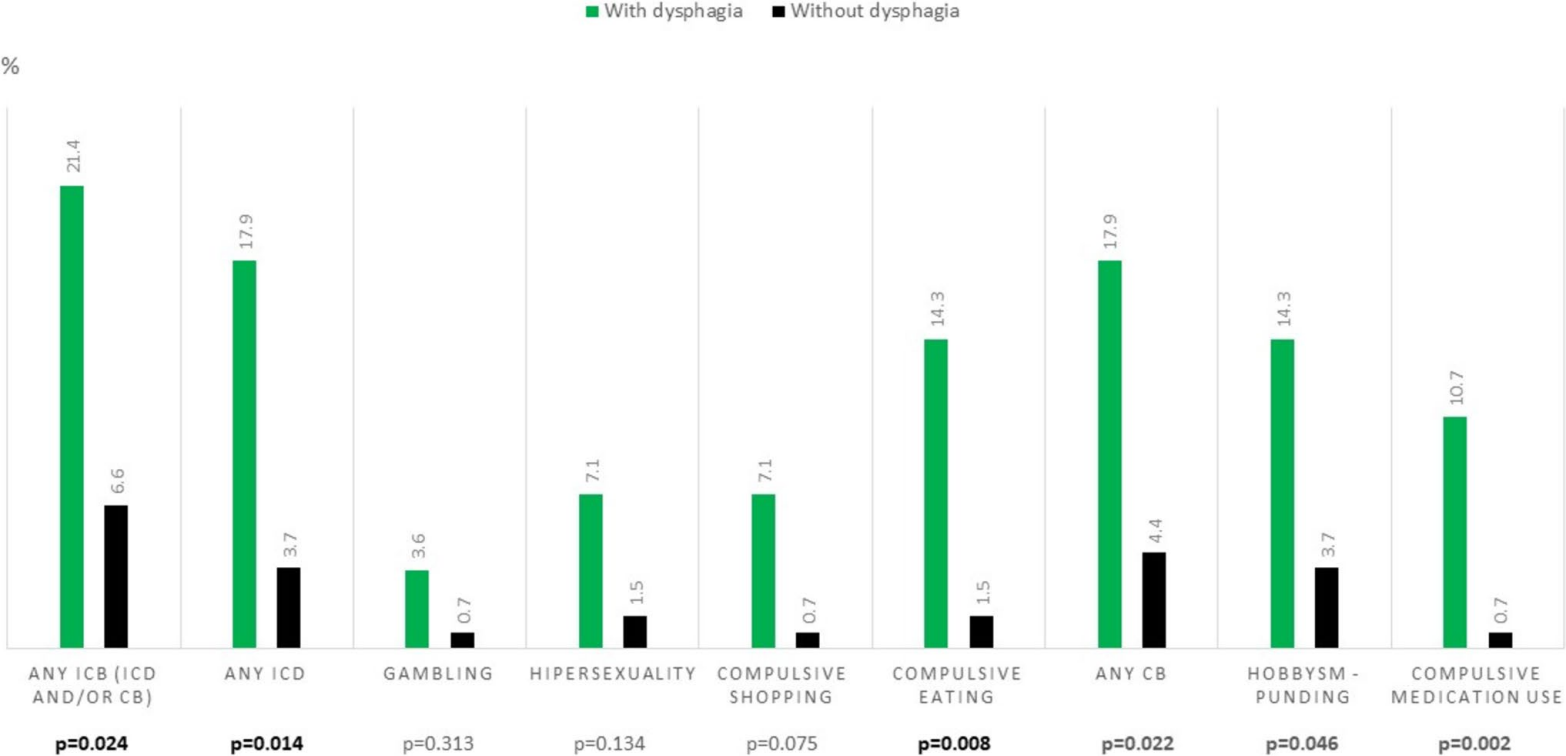
Percentage of PD patients with dysphagia (in green) vs without dysphagia (in black) suffering from impulse and/or compulsive symptoms. Fisher test was applied. CBs, compulsive behaviors; ICBs, impulsive-compulsive behaviors; ICDs, impulse control disorders

At V0 and comparing patients with vs without dysphagia, a higher score on the UPDRS-IV (1.4 ± 1.6 vs 0.9 ± 1.4; *p* = 0.014), FOGQ (3.3 ± 3.8 vs 1.9 ± 2.9; *p* = 0.010), NMSS (62.2 ± 35.3 vs 35.7 ± 30.8; *p* < 0.0001), BDI-II (10.9 ± 7.8 vs 7.8 ± 7.1; *p* = 0.019), QUIP-RS (7.8 ± 14 vs 2.3 ± 4.9; *p* = 0.007), VASF – physical (3.6 ± 2.7 vs 2.4 ± 2.7; *p* = 0.007), VASF – mental (3.4 ± 2.8 vs 1.8 ± 2.3; *p* = 0.001), and PDQ-39SI (23.2 ± 14.4 vs 12.6 ± 11.3; *p* < 0.0001) and a lower score on the PDSS (98.8 ± 30.2 vs 120.8 ± 25.7; *p* < 0.0001) and ADLS (87.5 ± 11.6 vs 92.8 ± 8.2; *p* = 0.028) were detected in those patients with dysphagia (Table 1). Specifically, FOG (36.1% vs 18.2%; *p* = 0.021), falls (22.2% vs 6.2%; *p *= 0.007), drooling (55.6% vs 29.7%; *p* = 0.015), and to have any ICB (21.4% vs 6.6%; *p* = 0.024) were more frequent in patients with dysphagia. Both ICDs and CBs were associated with dysphagia with compulsive eating the most significant ICB associated with dysphagia (14.3% in patients with dysphagia vs 1.5% in those without dysphagia; *p* = 0.008) (Fig. 1). Dysphagia correlated moderately with drooling (r = 0.348; *p* < 0.0001) but not with speech problems (r = −0.115; *p* = 0.445) or hypomimia (r = −0.098; *p* = 0.515). In a regression binary model (dysphagia at baseline as dependent variable), the factors identified as independently associated with dysphagia were a worse quality of sleep (PDSS; OR = 0.974; *p* = 0.005) and a greater ICB (QUIP-RS; OR = 1.066; *p* = 0.014) and NMS burden (NMSS; OR = 1.016; p = 0.021) (Table 2). The association between the QUIP-RS and dysphagia was maintained after adjusting to be receiving a dopamine agonist (QUIP-RS; OR = 1.073; *p* = 0.008). Specifically, to have any ICD as a whole (OR = 3.848; 95% CI 1.246 – 11.887; *p* = 0.019), any ICD (OR = 5.696; 95% CI 1.527 – 21.245; *p* = 0.010), any CB (OR = 4.746; 95% CI 1.337 – 16.848; *p* = 0.016), compulsive eating (OR = 11.25; 95% CI 1.951 – 64.871; *p* = 0.007), hobbyism-punding (OR = 4.4; 95% CI 1.002 – 11.575; *p* = 0.007), and DDS (OR = 13.364; 95% CI 1.347 – 132.553; *p* = 0.027) were associated with dysphagia, but none were after adjusting to covariates of the model. At V5 (*N* = 116), the QUIP-RS was associated again to dysphagia (OR = 1.042; 95% CI 1.001 – 1.086; *p* = 0.046), but it was not significant after adjustment to covariates (*p* = 0.274).

**Table 1:** Disease related characteristics, motor and non-motor symptoms, autonomy for activities of daily living and quality of life in PD patients with vs without dysphagia at baseline (*N* = 184)

	All cohort (*N* = 184)	Without dysphagia (*N* = 148)	With dysphagia (*N* = 36)	*p*
Age	62.3 ± 8.3	62.2 ± 8.4	62.8 ± 8.1	0.702
Males (%)	56.8	58.1	52.8	0.346
Disease duration (years)	1.3 ± 0.7	1.3 ± 0.7	1.1 ± 0.8	0.207
L-dopa daily dose (mg)	317 ± 247.4	317.1 ± 252.5	316.6 ± 228.1	0.753
Weight (kgs)	75.8 ± 13.5	75.7 ± 14.2	76 ± 10.9	0.660
Motor phenotype (%):				0.777
- Tremoric dominant	58.7	57.5	63.9	
- PIGD	28.3	29.7	22.2	
- Indeterminate	13	12.8	13.9	
Hoehn & Yahr – OFF	2 [1.5,2]	2 [1.5,2]	2 [1.5,2]	0.440
- Stage 3 – 5 (%)	2.5	3.1	0	0.299
UPDRS-III—OFF	19.1 ± 9.1	19.2 ± 9.4	18.8 ± 7.6	0.814
UPDRS-IV	1 ± 1.4	0.9 ± 1.4	1.4 ± 1.6	0.014
- Motor fluctuations (%)	8.7	6.8	16.7	0.060
FOGQ	2.2 ± 3.1	1.9 ± 2.9	3.3 ± 3.8	0.010
- FOG (%)	21.7	18.2	36.1	0.021
- Falls (%)	9.4	6.2	22.2	0.007
MMSE	29.3 ± 1	29.4 ± 1	29.1 ± 1.1	0.242
PD-CRS total score	90.7 ± 14.9	90.8 ± 15.1	90.6 ± 14.3	0.858
PD-CSR FS sub-score	62.8 ± 13.5	62.7 ± 13.6	63.4 ± 13.3	0.806
PD-CRS PC sub-score	27.9 ± 3.5	28.1 ± 3.3	27.2 ± 4.3	0.761
NMSS	40.9 ± 33.4	35.7 ± 30.8	62.2 ± 35.3	< 0.0001
- Drooling (%)	34.8	29.7	55.6	0.004
- Speech problems (%)	39	38	42.9	0.368
- Hypomimia (%)	76.1	81.2	64.3	0.192
BDI-II	8.4 ± 7.4	7.8 ± 7.1	10.9 ± 7.8	0.019
- Major depression (%)	16.3	14.2	25	0.096
NPI	5.3 ± 6.8	4.7 ± 6.1	7.7 ± 8.5	0.063
QUIP-RS	3.3 ± 7.6	2.3 ± 4.9	7.8 ± 14	0.007
- Any ICB (ICD or CB) (%)	9.1	6.6	21.4	0.024
- Any ICD (%)	6.1	3.7	17.9	0.014
- Any CB (%)	6.7	4.4	17.9	0.022
PDSS	116.6 ± 27.9	120.9 ± 25.7	98.8 ± 30.2	< 0.0001
VAS-PAIN	2.7 ± 2.9	2.6 ± 2.8	3 ± 3.1	0.467
VASF − physical	2.6 ± 2.7	2.4 ± 2.7	3.6 ± 2.7	0.007
VASF – mental	2.1 ± 2.5	1.8 ± 2.3	3.4 ± 2.8	0.001
ADLS	91.1 ± 9.1	92 ± 8.2	87.5 ± 11.6	0.028
PDQ-39SI	14.7 ± 12.6	12.6 ± 11.3	23.2 ± 14.4	< 0.0001
EUROHIS-QOL8	3.8 ± 0.6	3.9 ± 0.5	3.7 ± 0.6	0.050
PQ-10	7.4 ± 1.6	7.4 ± 1.6	7.3 ± 1.6	0.424

The results represent percentages, mean ± SD or median [p25, p75]. Chi-squared, Fisher and ANOVA test were applied. Data about H&Y and UPDRS-III are during the OFF state (first thing in the morning without taking medication in the previous 12 h)

*ADLS* Schwab and England Activities of daily living Scale), *BDI-II* Beck Depression Inventory-II, *CB* compulsive behavior, *FOGQ* Freezing of Gait Questionnaire, *FOG* freezing of gait, *ICB* impulse control behavior, *ICD* impulse control disorder, *NMSS* Non-Motor Symptoms Scale, *NPI* Neuropsychiatric Inventory, *PD* Parkinson´s disease, *PD-CRS* Parkinson’s Disease Cognitive Rating Scale, *PDSS* Parkinson’s Disease Sleep Scale, *PIGD* Postural Inestability Gait Dificulty, *QUIP-RS* Questionnaire for Impulsive-Compulsive Disorders in Parkinson’s Disease-Rating Scale, *UPDRS* Unified Parkinson’s Disease Rating Scale, *VAFS* Visual Analog Fatigue Scale, *VAS-Pain* Visual Analog Scale-Pain

**Table 2:** Independent factors associated with dysphagia in PD patients at baseline (*N* = 184)

Variables at baseline	OR^a^	OR^b^	Hosmer – Lemeshow test	R^2^	95% IC^a^	95% IC^b^	p^a^	p^b^
PDSS	0.976	0.974	0.59	0.35	0.96 – 0.99	0.96 – 0.99	< 0.0001	0.005
QUIP-RS	1.074	1.066			1.03 – 1.13	1.02 – 1.12	0.003	0.014
NMSS	1.021	1.016			1.01 – 1.03	1.01 – 1.03	< 0.0001	0.021

Dependent variable: Dysphagia at baseline. OR and 95% IC are shown. a, univariate analysis; b, multivariate analysis. The model was adjusted to age, gender, disease duration and LEDD. *LEDD* levodopa equivalent daily dose, *NMSS* Non-Motor Symptoms Scale, *PDSS* Parkinson´s Disease Sleep Scale, *QUIP-RS* Questionnaire for Impulsive-Compulsive Disorders in Parkinson's Disease-Rating Scale

**Fig. 2:**
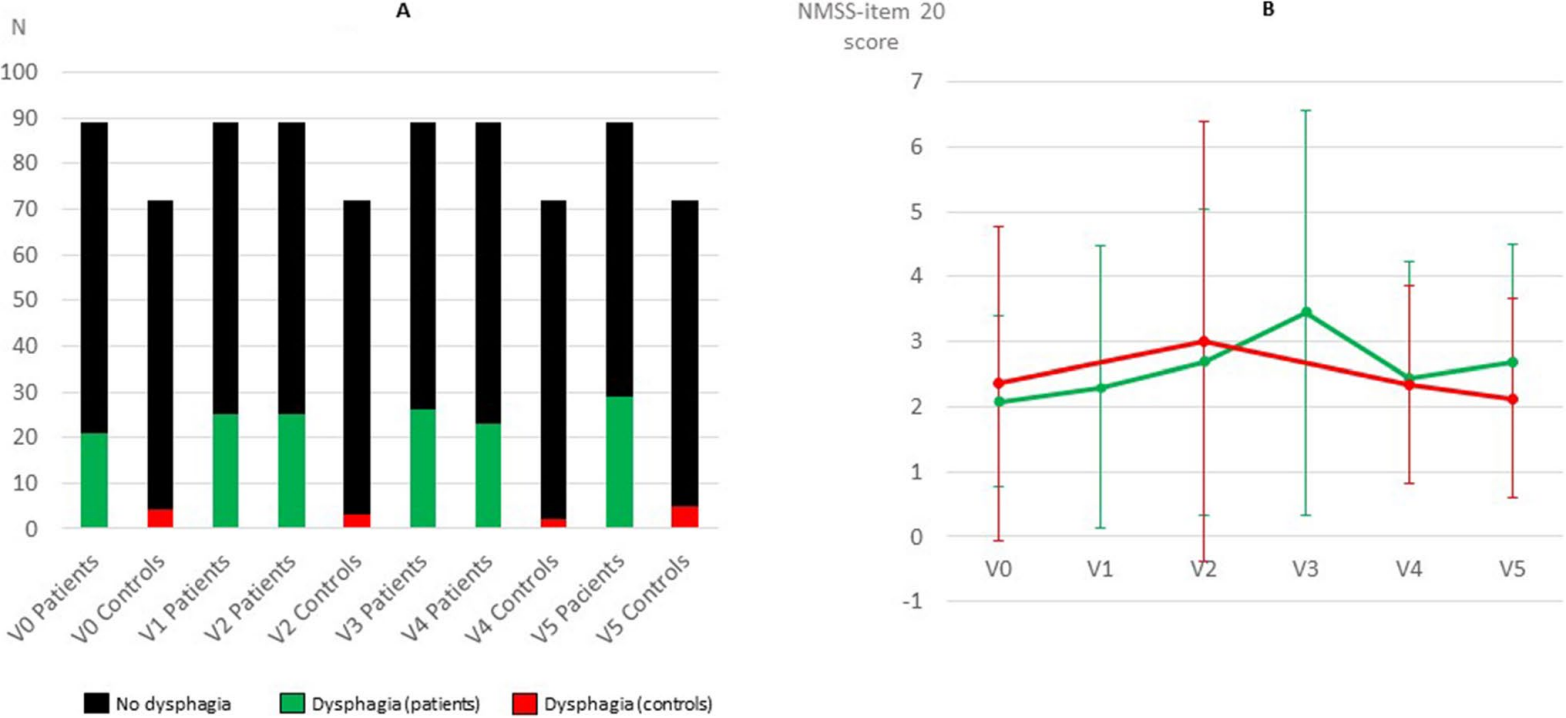
**A.** Number of PD patients and/or controls with and without dysphagia at V0, V1, V2, V3, V4 and V5. At V0, V2, V4 and V5, the percentage of dysphagia was significantly higher in patients than in controls (*p* < 0.0001 for all analysis). The Chi-square test was applied. **B.** NMSS-item 20 score in PD patients with dysphagia at V0, V1, V2, V3, V4 and V5 and in controls at V0, V2, V4 and V5. Mean and standard deviation are shown. No significant differences (*p* > 0.05) were detected between patients and controls in any visit or between the different visits throughout the follow-up in either patients and controls. T-Student and/or General linear model (GLM) repeated measure was adjusting to age, gender, disease duration and levodopa equivalent daily dose at each visit

In the follow-up analysis (all data in all visits for all participants, from V0 to V5), 89 patients (61 ± 8.6 years old; 53.9% males) and 72 controls (63.1 ± 6.7 years old; 50% males) were included. Dysphagia was clearly more frequent in PD patients than controls (*p* < 0.0001 at V0, V2, V4 and V5): 23.6% at V0, 28.1% at V1, 28.1% at V2, 29.2% at V3, 25.8% at V4, and 32.6% at V5 in patients; 5.6% at V0, 4.2% at V2, 2.8% at V4, and 6.9% at V5 in controls (Fig. 2A). Mean score of the NMSS-item 20 was significantly higher in PD patients than controls (*p* < 0.0001 in all visits): V0, 0.6 ± 1.2 vs 0.2 ± 1.2; V2, 0.7 ± 1.6 vs 0.2 ± 1.1; V4, 0.7 ± 1.5 vs 0.1 ± 0.5; V5, 0.7 ± 1.5 vs ± 0.2 ± 0.7. However, no statistically significant differences (*p* ≥ 0.05) between PD patients and controls in the NMSS-item 20 score were detected in any of the visits when only subjects with dysphagia were included (Fig. 2B). No differences (Cohen´s d formula < 0.2 and *p* > 0.05 for all analysis) in the mean NMSS-item 20 score were detected between the different visits (from V0 to V5; from V0 to V1; from V1 to V2; from V2 to V3; from V3 to V4; from V4 to V5) throughout the follow-up neither in patients nor controls. Relevant dysphagia burden was detected only in 1.1%, 6.6%, 3.3%, 6.7%, 4.5%, and 3.3% of the patients at V0, V1, V2, V3, V4, and V5, respectively.

## Discussion

The present study observed that dysphagia is frequent in early PD patients (≤ 2 years of disease duration), affecting about 1 out of 4 patients. However, dysphagia burden was low and did not progress over time, like in controls. Although many factors were associated with dysphagia in early stages of PD, only a worse quality of sleep and a greater ICB and NMS burden were independent factor associated with dysphagia. To our knowledge, this is the first time that an association between dysphagia and ICBs have been described.

Throughout the 5 years of follow-up of our cohort, the prevalence of dysphagia was clearly higher in PD patients (from 23.6% at baseline to 32.6% after 5 years of follow-up) than in controls (from 2.8% to 6.9%) and considerably high overall considering the fact that they had less than 2 years from symptoms onset at baseline. PD is one of the important etiologies of dysphagia, showing a pooled prevalence of 35% and up to 82% using subjective measures and objective measures, respectively [2]. Differences in the methodology including dysphagia definition and the characteristics of PD populations explain the great prevalence range. Since dysphagia is considered a red flag that would suggest atypical parkinsonism [1], it is important to parse out how frequent dysphagia is in PD at diagnosis. Very little information has been published about this. Only 2 out of 58 studies about dysphagia in PD were conducted in PD patients with a disease duration ≤ 2 years [3, 9, 10]. Our finding of a dysphagia prevalence of 19.6% (36 out of 184 patients) at baseline agrees with the 20.1% reported by Malek et al. [10] in 1746 patients that had been diagnosed within the preceding 3.5 years (mean disease duration of 1.3 years) and 20.1% (vs 3% in controls) detected by Khoo et al. [19] in 159 patients with a median disease duration of 4.4 months. However, this was higher than the 12.3% reported by Polychronis et al. [9] in 398 PD patients with a mean disease duration of 0.5 years. As in our study, dysphagia was recorded in all these studies [9, 10, 19] according to a question of a general scale, the SCOPA-AUT [9, 10] and the NMSQuest [19], and not with objective methods. We used a question (item 20) of the NMSS and results must be interpreted with caution because the prevalence could be higher if a more specific scale validated for the detection of dysphagia or especially objective methods were used [20]. In fact, and surprisingly, 0 out of 41 newly diagnosed PD patients had “difficulty swallowing food or drinking or problems with choking” in a study in which the NMSQuest was used [21]. Comparing to all studies conducted in early PD patients, we demonstrated that although dysphagia could be frequent even in the first 2 years from symptoms onset and is clearly more common than in controls, relevant dysphagia is rare, and dysphagia burden doesn’t progress over time after a 5-year follow-up. Nonetheless, this could be a cue to differentiate parkinsonism with dysphagia is due to PD vs. an atypical parkinsonism [22].

Some factors have been identified to be associated with dysphagia in PD such as older age, male sex, lower body mass index, longer disease duration, higher H&Y stage and levodopa equivalent daily dose (LEDD), PIGD subtype, severe motor symptoms, cognitive impairment, drooling, higher levels of depression and anxiety, and lower QoL [3–5]. Many of them are related to the progression of the disease and in fact, the prevalence of dysphagia is higher in advanced PD, reaching up to more than 80% [23]. However, despite our analysis being conducted in early PD patients (mean disease duration 1.3 ± 0.7) with 97.5% in the stage 2 of H&Y, significant differences between patients with and without dysphagia were observed in motor aspects (UPDRS-IV, FOG and falls), NMS including drooling, mood (BDI-II), ICBs (QUIP-RS), sleep (PDSS), and fatigue (VAFS), disability (ADLS), and PD related QoL (PDQ-39SI). Of them, only a worse quality of sleep and a greater ICB and NMS burden were independent factors associated with dysphagia. Interestingly, Marano et al. [23] observed that excessive daytime sleepiness was strongly related to the development of swallowing impairment after a 3-year follow-up in a retrospective study conducted in early-stage PD patients who were not on dopaminergic treatment at baseline. Drooling and dysphagia are related [3, 24], as we found, and can affect the sleep. Mean total sleep time (minutes) was 350.8 ± 79.3 in 5 PD patients with dysphagia compared to 406.4 ± 119.9 in 16 PD patients without dysphagia in a study conducted in 21 PD patients vs 18 controls with all-night sleep video-EEG and electromyography information collected [25]. Moreover, sleep is one of the items commonly affected in studies analyzing the QOL related to swallowing [26].

NMS burden was also related to dysphagia in our cohort. A greater burden of NMS in patients with dysphagia could suggest the presence of a phenotype with more non-motor involvement. Additionally, the presence of dysphagia could be a useful indication to investigate the presence of other NMS. The pathophysiology of dysphagia is complex and many areas of the brain and even in the peripheral nervous system could be involved [27], which could explain the greater NMS burden that we found associated with dysphagia. On the other hand, our limited knowledge on the relationship between dysphagia and ICB is more difficult to explain. Importantly, this is the first time described, but probably, the first time explored as well [28]. As we did not collected information to classify the patient’s swallow difficulty (i.e., oral phase vs pharyngeal phase vs esophageal phase), all possible suggestions are purely speculative. Dysphagia is also associated with depression and anxiety due to (i) the severity and impact of the symptom itself, (ii) the underlying cause, and/or (iii) inappropriate responses to swallowing problems leading to subsequent impulsive eating behaviors or other maladaptive behaviors, among other reasons [29]. To think of a frontal lobe dysfunction as a cause [30], we did not observe differences in cognitive function between PD patients with vs without dysphagia, like in some studies [3]. Interestingly, in the ICARUS study [31], patients who were ICD-positive at study baseline had more severe NMS (including mood and sexual function) and depression, as well as had poorer sleep quality and reduced PD-related QoL compared with those who were ICD-negative, like in our cohort with those patients with dysphagia. This possible relationship between ICBs and dysphagia should be properly explored. In favor of it, a significant association with the QUIP-RS was obtained again after the 5-year follow-up in this cohort of early PD patient. When considering all the cohort, the QUIP-RS was associated to dysphagia as well (data not shown; *N* = 691; OR = 1.027; 95% CI 1.003 – 1.051; *p* = 0.027) after adjustment to age, gender, disease duration, H&Y stage, dopamine agonist treatment, UPDRS-IV, NMSS, and PDSS. Finally, and regarding QoL, patients with dysphagia had a worse QoL according to the PDQ-39, something previously reported in a meta-analysis [3]. This reinforces the importance of dysphagia as a disabling symptom for the patient [26].

The present study has limitations. The most important, as it has been mentioned, dysphagia was defined according to subjective methods using one question of a general questionnaire (NMSS). Secondarily, mechanisms involved in dysphagia in these patients were not analyzed. However, this is frequent in the literature applying post-hoc analysis, like ours, in big cohorts [9, 10]. Second, for some analysis comparing groups (e.g., ICB), the sample was small. Third, our findings may not be applicable to all PD patients in the community clinical setting be-cause PD patients enrolled in the COPPADIS study represent a selected population with less disability at baseline than the general population (e.g., no older than 75 years old, not being under a second line therapy, etc.). Finally, other markers (neuroimaging, genetic, sleep study, etc.) were not used to explain the association between dysphagia and other variables (e.g., ICD). On the other hand, and as strengths, this is a 5-year follow-up study with a comparing control group in which extensive information about sociodemographic aspects, motor and NMS, disability and QoL has been compared regarding dysphagia. For the first time, a relationship between the QUIP-RS score and dysphagia has been reported.

In conclusion, dysphagia was frequent in early PD patients and clearly higher compared to controls. However, it was minor and did not progress over time. Sleep, ICB, and non-motor symptoms burden were related to dysphagia. More studies specifically designed to analyze the relationship between dysphagia with other symptoms using subjective combined with objective methods at the same time are required. Replication of this association between dysphagia and ICD is required.

## Supplementary Information

Below is the link to the electronic supplementary material.

Supplementary file1 (DOCX 60 KB)

Supplementary file2 (PDF 383 KB)

Supplementary file3 (PDF 348 KB)

Supplementary file4 (PDF 299 KB)

## Data Availability

The protocol and the statistical analysis plan are available on request. Deidentified participant data are not available for legal and ethical reasons.
