# Supplementary material for: Dysphagia in Parkinson´s disease. A 5-year follow-up study
Source: Neurol Sci. 2025 Feb 19;46(6):2637–53. doi: 10.1007/s10072-025-08027-8 (PMC12084275; doi:10.1007/s10072-025-08027-8)
Supplement: Supplementary file 1 — Supplementary file1 (DOCX 60 KB) [file 10072_2025_8027_MOESM1_ESM.docx]

**COPPADIS STUDY GROUP**

Adarmes AD, Almeria M, Alonso Losada MG, Alonso Cánovas A, Alonso Frech F, Alonso Redondo R, Álvarez I, Álvarez Sauco M, Aneiros Díaz A, Arnáiz S, Arribas S, Ascunce Vidondo A, Aguilar M, Ávila MA, Bernardo Lambrich N, Bejr-Kasem H, Blázquez Estrada M, Botí M, Borrue C, Buongiorno MT, Cabello González C, Cabo López I, Caballol N, Cámara Lorenzo A, Canfield Medina H, Carabajal Pendón E, Carrillo F, Carrillo Padilla FJ, Casas E, Catalán MJ, Clavero P, Cortina Fernández A, Cosgaya M, Cots Foraster A, Crespo Cuevas A, Cubo E, de Deus Fonticoba T, de Fábregues-Boixar O, Díez-Fairen M, Dotor García-Soto J, Erro E, Escalante S, Estelrich Peyret E, Fernández Guillán N, Gámez P, Gallego M, García Caldentey J, García Campos C, García Díez C, García Moreno JM, Gastón I, Gómez Garre MP, Gómez Mayordomo V, González Aloy J, González-Aramburu I, González Ardura J, González García B, González Palmás MJ, González Toledo GR, Golpe Díaz A, Grau Solá M, Guardia G, Hernández Vara J, Horta-Barba A, Idoate Calderón D, Infante J, Jesús S, Kulisevsky J, Kurtis M, Labandeira C, Labrador MA, Lacruz F, Lage Castro M, Lastres Gómez S, Legarda I, López Ariztegui N, López Díaz LM, López Domínguez D, López Manzanares L, López Seoane B, Lucas del Pozo S, Macías Y, Mata M, Martí Andres G, Martí MJ, Martínez Castrillo JC, Martinez-Martin P, McAfee D, Meitín MT, Mendoza Plasencia Z, Menéndez González M, Méndez del Barrio C, Mir P, Miranda Santiago J, Morales Casado MI, Moreno Diéguez A, Muro García I, Nogueira V, Novo Amado A, Novo Ponte S, Ordás C, Pagonabarraga J, Pareés I, Pascual-Sedano B, Pastor P, Pérez Fuertes A, Pérez Noguera R, Planas-Ballvé A, Planellas L, Prats MA, Prieto Jurczynska C, Puente V, Pueyo Morlans M, Puig Daví A, Redondo Rafales N, Rodríguez Méndez L, Rodríguez Pérez AB, Roldán F, Ruíz De Arcos M, Ruíz Martínez J, Sánchez Alonso P, Sánchez-Carpintero M, Sánchez Díez G, Sánchez Rodríguez A, Santacruz P, Santos García D, Segundo Rodríguez JC, Seijo M, Sierra Peña M, Solano Vila B, Suárez Castro E, Tartari JP, Valero C, Vargas L, Vela L, Villanueva C, Vives B.

| **Name (Last Name, First Name)** | **Location** | **Role** | **Contribution** |
| --- | --- | --- | --- |
| Astrid Adarmes, Daniela | Hospital Universitario Virgen del Rocío, Sevilla, Spain | Site investigator | Evaluation of participants and/or data management |
| Almeria, Marta | Hospital Universitari Mutua de Terrassa, Terrassa, Barcelona, Spain | Site investigator | Neuropsychologist; evaluation of participants |
| Alonso Losada, Maria Gema | Hospital Álvaro Cunqueiro, Complejo Hospitalario Universitario de Vigo (CHUVI), Vigo, Spain | Site investigator / PI | Coordination at the center  Evaluation of participants and/or data management |
| Alonso Cánovas, Araceli | Hospital Universitario Ramón y Cajal, Madrid, Spain | Site investigator | Evaluation of participants and/or data management |
| Alonso Frech, Fernando | Hospital Universitario Clínico San Carlos, Madrid, Spain | Site investigator | Evaluation of participants and/or data management |
| Alonso Redondo, Ruben | Hospital Universitario Lucus Augusti (HULA), Lugo, Spain | Site investigator / PI | Coordination at the center  Evaluation of participants and/or data management |
| Aneiros Díaz, Ángel | Complejo Hospitalario Universitario de Ferrol (CHUF), Ferrol, A Coruña, Spain | Site investigator / PI | Coordination at the center  Evaluation of participants and/or data management |
| Álvarez, Ignacio | Hospital Universitari Mutua de Terrassa, Terrassa, Barcelona, Spain | Site investigator | Evaluation of participants and/or data management |
| Álvarez Sauco, María | Hospital General Universitario de Elche, Elche, Spain | Site investigator / PI | Coordination at the center  Evaluation of participants and/or data management |
| Arnáiz, Sandra | Complejo Asistencial Universitario de Burgos, Burgos, Spain | Site investigator | Evaluation of participants and/or data management |
| Arribas, Sonia | Hospital Universitari Mutua de Terrassa, Terrassa, Barcelona, Spain | Site investigator | Neuropsychologist; evaluation of participants |
| Ascunce Vidondo, Arancha | Complejo Hospitalario de Navarra, Pamplona, Spain | Site investigator | Evaluation of participants and/or data management |
| Aguilar, Miquel | Hospital Universitari Mutua de Terrassa, Terrassa, Barcelona, Spain | Site investigator | Evaluation of participants and/or data management |
| Ávila Rivera, Maria Asunción | Consorci Sanitari Integral, Hospital General de L´Hospitalet, L´Hospitalet de Llobregat, Barcelona, Spain | Site investigator / PI | Coordination at the center  Evaluation of participants and/or data management |
| Bernardo Lambrich, Noemí | Hospital de Tortosa Verge de la Cinta (HTVC), Tortosa, Tarragona, Spain | Site investigator | Evaluation of participants and/or data management |
| Bejr-Kasem, Helena | Hospital de Sant Pau, Barcelona, Spain | Site investigator | Evaluation of participants and/or data management |
| Blázquez Estrada, Marta | Hospital Universitario Central de Asturias, Oviedo, Spain | Site investigator | Evaluation of participants and/or data management |
| Botí González, Maria Ángeles | Hospital Universitari Mutua de Terrassa, Terrassa, Barcelona, Spain | Site investigator | Neuropsychologist; evaluation of participants |
| Borrué, Carmen | Hospital Infanta Sofía, Madrid, Spain | Site investigator / PI | Coordination at the center  Evaluation of participants and/or data management |
| Buongiorno, Maria Teresa | Hospital Universitari Mutua de Terrassa, Terrassa, Barcelona, Spain | Site investigator (until 2024) | Nurse study coordinator |
| Cabello González, Carolina | Complejo Hospitalario de Navarra, Pamplona, Spain | Site investigator | Scheduling of evaluations |
| Cabo López, Iria | Complejo Hospitalario Universitario de Pontevedra (CHOP), Pontevedra, Spain | Site investigator / PI | Coordination at the center  Evaluation of participants and/or data management |
| Caballol, Nuria | Consorci Sanitari Integral, Hospital Moisés Broggi, Sant Joan Despí, Barcelona, Spain. | Site investigator / PI | Coordination at the center  Evaluation of participants and/or data management |
| Cámara Lorenzo, Ana | Hospital Clínic de Barcelona, Barcelona, Spain | Site investigator | Nurse study coordinator |
| Canfield Medina, Héctor | Complejo Hospitalario Universitario de Ferrol (CHUF), Ferrol, A Coruña, Spain | Site investigator | Evaluation of participants and/or data management |
| Carabajal Pendón, Estefanía | Hospital La Princesa, Madrid, Spain | Site investigator (from MAR/23) | Evaluation of participants and/or data management |
| Carrillo, Fátima | Hospital Universitario Virgen del Rocío, Sevilla, Spain | Site investigator | Evaluation of participants and/or data management |
| Carrillo Padilla, Francisco José | Hospital Universitario de Canarias, San Cristóbal de la Laguna, Santa Cruz de Tenerife, Spain | Site investigator / PI | Coordination at the center  Evaluation of participants and/or data management |
| Casas, Elena | Complejo Asistencial Universitario de Burgos, Burgos, Spain | Site investigator | Evaluation of participants and/or data management |
| Catalán, Maria José | Hospital Universitario Clínico San Carlos, Madrid, Spain | Site investigator / PI | Coordination at the center  Evaluation of participants and/or data management |
| Clavero, Pedro | Complejo Hospitalario de Navarra, Pamplona, Spain | Site investigator | Evaluation of participants and/or data management |
| Cortina Fernández, A | Complejo Hospitalario Universitario de Ferrol (CHUF), Ferrol, A Coruña, Spain | Site investigator | Coordination of blood extractions |
| Cosgaya, Marina | Hospital Clínic de Barcelona, Barcelona, Spain | Site investigator | Evaluation of participants and/or data management |
| Cots Foraster, Anna | Institut d'Assistència Sanitària (IAS) - Instituí Cátala de la Salud. Girona, Spain | Site investigator | Evaluation of participants and/or data management |
| Crespo Cuevas, Ane | Hospital del Mar, Barcelona, Spain. | Site investigator | Evaluation of participants and/or data management |
| Cubo, Esther | Complejo Asistencial Universitario de Burgos, Burgos, Spain | Site investigator / PI | Coordination at the center  Evaluation of participants and/or data management |
| De Deus Fonticoba, Teresa | Complejo Hospitalario Universitario de Ferrol (CHUF), Ferrol, A Coruña, Spain | Site investigator | Nurse study coordinator  Evaluation of participants and/or data management |
| De Fábregues-Boixar, Oriol | Hospital Universitario Vall d´Hebron, Barcelona, Spain | Site investigator / PI | Coordination at the center  Evaluation of participants and/or data management |
| Díez Fairen, M | Hospital Universitari Mutua de Terrassa, Terrassa, Barcelona, Spain | Site investigator | Evaluation of participants and/or data management |
| Dotor García-Soto, Julio | Hospital Universitario Virgen Macarena, Sevilla, Spain | Site investigator / PI | Evaluation of participants and/or data management |
| Erro, Elena | Complejo Hospitalario de Navarra, Pamplona, Spain | Site investigator | Evaluation of participants and/or data management |
| Escalante, Sonia | Hospital de Tortosa Verge de la Cinta (HTVC), Tortosa, Tarragona, Spain | Site investigator / PI | Coordination at the center  Evaluation of participants and/or data management |
| Estelrich Peyret, Elena | Institut d'Assistència Sanitària (IAS) - Instituí Cátala de la Salud. Girona, Spain | Site investigator | Evaluation of participants and/or data management |
| Fernández Guillán, Noelia | Complejo Hospitalario Universitario de Ferrol (CHUF), Ferrol, A Coruña, Spain | Site investigator | Neuroimaging studies |
| Gámez, Pedro | Complejo Asistencial Universitario de Burgos, Burgos, Spain | Site investigator | Evaluation of participants and/or data management |
| Gallego, Mercedes | Hospital La Princesa, Madrid, Spain | Site investigator | Evaluation of participants and/or data management |
| García Caldentey, Juan | Centro Neurológico Oms 42, Palma de Mallorca, Spain | Site investigator / PI | Coordination at the center  Evaluation of participants and/or data management |
| García Campos, Cristina | Hospital Universitario Virgen Macarena, Sevilla, Spain | Site investigator | Evaluation of participants and/or data management |
| García Díez, Cristina | Complejo Hospitalario Universitario de Pontevedra (CHOP), Pontevedra, Spain | Site investigator (from MAY/22) | neuropsychologist; evaluation of participants |
| García Moreno, Jose Manuel | Hospital Universitario Virgen Macarena, Sevilla, Spain | Site investigator / PI (until MAR/21) | Coordination at the center  Evaluation of participants and/or data management |
| Gastón, Itziar | Complejo Hospitalario de Navarra, Pamplona, Spain | Site investigator / PI | Coordination at the center  Evaluation of participants and/or data management |
| Gómez Garre, María del Pilar | Hospital Universitario Virgen del Rocío, Sevilla, Spain | Site investigator | Genetic studies coordination |
| Gómez Mayordomo, Víctor | Hospital Clínico San Carlos, Madrid, Spain | Site investigator* | Evaluation of participants and/or data management |
| González Aloy, Javier | Institut d'Assistència Sanitària (IAS) - Instituí Cátala de la Salud. Girona, Spain | Site investigator | Evaluation of participants and/or data management |
| González Aramburu, Isabel | Hospital Universitario Marqués de Valdecilla, Santander, Spain | Site investigator | Evaluation of participants and/or data management |
| González Ardura, Jessica | Hospital Universitario Lucus Augusti (HULA), Lugo, Spain | Site investigator / PI (until FEB/21) | Evaluation of participants and/or data management |
| González García, Beatriz | Hospital La Princesa, Madrid, Spain | Site investigator | Nurse study coordinator |
| González Palmás, Maria Josefa | Complejo Hospitalario Universitario de Pontevedra (CHOP), Pontevedra, Spain | Site investigator | Evaluation of participants and/or data management |
| González Toledo, Gabriel Ricardo | Hospital Universitario de Canarias, San Cristóbal de la Laguna, Santa Cruz de Tenerife, Spain | Site investigator | Evaluation of participants and/or data management |
| Golpe Díaz, Ana | Complejo Hospitalario Universitario de Ferrol (CHUF), Ferrol, A Coruña, Spain | Site investigator | Laboratory analysis coordination |
| Grau Solá, Mireia | Consorci Sanitari Integral, Hospital Moisés Broggi, Sant Joan Despí, Barcelona, Spain | Site investigator | Evaluation of participants and/or data management |
| Guardia, Gemma | Hospital Universitari Mutua de Terrassa, Terrassa, Barcelona, Spain | Site investigator | Evaluation of participants and/or data management |
| Hernández Vara, Jorge | Hospital Universitario Vall d´Hebron, Barcelona, Spain | Site investigator / PI | Coordination at the center  Evaluation of participants and/or data management |
| Horta Barba, Andrea | Hospital de Sant Pau, Barcelona, Spain | Site investigator | Neuropsychologist; evaluation of participants |
| Idoate Calderón, Daniel | Complejo Hospitalario Universitario de Pontevedra (CHOP), Pontevedra, Spain | Site investigaor (until MAY/22) | neuropsychologist; evaluation of participants |
| Infante, Jon | Hospital Universitario Marqués de Valdecilla, Santander, Spain | Site investigator / PI | Coordination at the center  Evaluation of participants and/or data management |
| Jesús, Silvia | Hospital Universitario Virgen del Rocío, Sevilla, Spain | Site investigator | Evaluation of participants and/or data management |
| Kulisevsky, Jaime | Hospital de Sant Pau, Barcelona, Spain | Site investigator / PI | Coordination at the center  Evaluation of participants and/or data management |
| Kurtis, Mónica | Hospital Ruber Internacional, Madrid, Spain | Site investigator / PI | Coordination at the center  Evaluation of participants and/or data management |
| Labandeira, Carmen | Hospital Álvaro Cunqueiro, Complejo Hospitalario Universitario de Vigo (CHUVI), Vigo, Spain | Site investigator | Evaluation of participants and/or data management |
| Labrador Espinosa, Miguel Ángel | Hospital Universitario Virgen del Rocío, Sevilla, Spain | Site investigator | Neuroimaging data analysis |
| Lacruz, Francisco | Complejo Hospitalario de Navarra, Pamplona, Spain | Site investigator | Evaluation of participants and/or data management |
| Lage Castro, Melva | Complejo Hospitalario Universitario de Pontevedra (CHOP), Pontevedra, Spain | Site investigator | Evaluation of participants and/or data management |
| Lastres Gómez, Sonia | Complejo Hospitalario Universitario de Pontevedra (CHOP), Pontevedra, Spain | Site investigator | Neuropsychologist; evaluation of participants |
| Legarda, Inés | Hospital Universitario Son Espases, Palma de Mallorca, Spain | Site investigator / PI | Coordination at the center  Evaluation of participants and/or data management |
| López Ariztegui, Nuria | Complejo Hospitalario de Toledo, Toledo, Spain | Site investigator / PI | Evaluation of participants and/or data management |
| López Díaz, Luis Manuel | Hospital Da Costa de Burela, Lugo, Spain | Site investigator (until DEC/16) | Evaluation of participants and/or data management |
| López Domínguez, Daniel | Institut d'Assistència Sanitària (IAS) - Instituí Cátala de la Salud. Girona, Spain | Site investigator | Evaluation of participants and/or data management |
| López Manzanares, Lydia | Hospital La Princesa, Madrid, Spain | Site investigator / PI | Coordination at the center  Evaluation of participants and/or data management |
| López Seoane, Balbino | Complejo Hospitalario Universitario de Ferrol (CHUF), Ferrol, A Coruña, Spain | Site investigator | Neuroimaging studies |
| Lucas del Pozo, Sara | Hospital Universitario Vall d´Hebron, Barcelona, Spain | Site investigator | Evaluation of participants and/or data management |
| Macías, Yolanda | Fundación Hospital de Alcorcón, Madrid, Spain | Site investigator | Evaluation of participants and/or data management |
| Mata, Marina | Hospital Infanta Sofía, Madrid, Spain | Site investigator | Evaluation of participants and/or data management |
| Martí Andres, Gloria | Hospital Universitario Vall d´Hebron, Barcelona, Spain | Site investigator | Evaluation of participants and/or data management |
| Martí, Maria José | Hospital Clínic de Barcelona, Barcelona, Spain | Site investigator / PI | Coordination at the center  Evaluation of participants and/or data management |
| Martínez Castrillo, Juan Carlos | Hospital Universitario Ramón y Cajal, Madrid, Spain | Site investigator /PI | Coordination at the center  Evaluation of participants and/or data management |
| Martinez-Martin, Pablo | CIBERNED, Instituto de Salud Carlos III, Madrid, Spain | Collaborator in statistical and methods analysis | Methods and statistical reviewer |
| McAfee, Darrian | University of Pennsylvania, Philadelphia | Collaborator in english style | English style reviewer |
| Meitín, Maria Teresa | Hospital Da Costa de Burela, Lugo, Spain | Site investigator | Evaluation of participants and/or data management |
| Menéndez González, Manuel | Hospital Universitario Central de Asturias, Oviedo, Spain | Site investigator / PI | Coordination at the center  Evaluation of participants and/or data management |
| Méndez del Barrio, Carlota | Hospital Universitario Virgen del Rocío, Sevilla, Spain | Site investigator | Evaluation of participants and/or data management |
| Mendoza Plasencia, Zebenzui | Hospital Universitario de Canarias, San Cristóbal de la Laguna, Santa Cruz de Tenerife, Spain | Site investigator | Evaluation of participants and/or data management |
| Mir, Pablo | Hospital Universitario Virgen del Rocío, Sevilla, Spain | Site investigator / PI | Coordination at the center  Evaluation of participants and/or data management |
| Miranda Santiago, Javier | Complejo Asistencial Universitario de Burgos, Burgos, Spain | Site investigator | Evaluation of participants and/or data management |
| Morales Casado, Maria Isabel | Complejo Hospitalario de Toledo, Toledo, Spain. | Site investigator | Evaluation of participants and/or data management |
| Moreno Diéguez, Antonio | Complejo Hospitalario Universitario de Ferrol (CHUF), Ferrol, A Coruña, Spain | Site investigator | Neuroimaging studies |
| Muro García, Inés | Hospital La Princesa, Madrid, Spain | Site investigator (from MAR/23) | Evaluation of participants and/or data management |
| Nogueira, Víctor | Hospital Da Costa de Burela, Lugo, Spain | Site investigator / PI (until 2022)** | Coordination at the center  Evaluation of participants and/or data management |
| Novo Amado, Alba | Complejo Hospitalario Universitario de Ferrol (CHUF), Ferrol, A Coruña, Spain | Site investigator | Neuroimaging studies |
| Novo Ponte, Sabela | Hospital Universitario Puerta de Hierro, Madrid, Spain. | Site investigator | Evaluation of participants and/or data management |
| Ordás, Carlos | Hospital Rey Juan Carlos, Madrid, Spain, Madrid, Spain. | Site Investigator | Evaluation of participants and/or data management |
| Pagonabarraga, Javier | Hospital de Sant Pau, Barcelona, Spain | Site investigator | Evaluation of participants and/or data management |
| Pareés, Isabel | Hospital Ramón y Cajal, Madrid, Spain | Site investigator | Evaluation of participants and/or data management |
| Pascual-Sedano, Berta | Hospital de Sant Pau, Barcelona, Spain | Site Investigator | Evaluation of participants and/or data management |
| Pastor, Pau | Hospital Universitari Mutua de Terrassa, Terrassa, Barcelona, Spain | Site investigator | Evaluation of participants and/or data management |
| Pérez Fuertes, Aída | Complejo Hospitalario Universitario de Ferrol (CHUF), Ferrol, A Coruña, Spain | Site investigator | Blood analysis |
| Pérez Noguera, Rafael | Hospital Universitario Virgen Macarena, Sevilla, Spain | Site investigator | Evaluation of participants and/or data management |
| Planas-Ballvé, Ana | Consorci Sanitari Integral, Hospital Moisés Broggi, Sant Joan Despí, Barcelona, Spain | Site investigator | Evaluation of participants and/or data management |
| Planellas, Lluís | Hospital Clínic de Barcelona, Barcelona, Spain | Site investigator (until DEC/19) | Evaluation of participants and/or data management |
| Prats, Marian Ángeles | Institut d'Assistència Sanitària (IAS) - Instituí Cátala de la Salud. Girona, Spain | Site investigator | Evaluation of participants and/or data management |
| Prieto Jurczynska, Cristina | Hospital Rey Juan Carlos, Madrid, Spain, Madrid, Spain | Site investigator / PI | Coordination at the center  Evaluation of participants and/or data management |
| Puente, Víctor | Hospital del Mar, Barcelona, Spain | Site investigator / PI | Coordination at the center  Evaluation of participants and/or data management |
| Pueyo Morlans, Mercedes | Hospital Universitario de Canarias, San Cristóbal de la Laguna, Santa Cruz de Tenerife, Spain | Site investigator | Evaluation of participants and/or data management |
| Puig Daví, Arnau | Hospital de Sant Pau, Barcelona, Spain | Site einvestigator | Evaluation of participants and/or data management |
| Redondo, Nuria | Hospital La Princesa, Madrid, Spain | Site Investigator | Evaluation of participants and/or data management |
| Rodríguez Méndez, Luisa | Complejo Hospitalario Universitario de Ferrol (CHUF), Ferrol, A Coruña, Spain | Site investigator | Blood analysis |
| Rodríguez Pérez, Amparo Belén | Hospital General Universitario de Elche, Elche, Spain | Site investigator | Evaluation of participants and/or data management |
| Roldán, Florinda | Hospital Universitario Virgen del Rocío, Sevilla, Spain | Site investigator | Neuroimaging studies |
| Ruíz de Arcos, María | Hospital Universitario Virgen Macarena, Sevilla, Spain. | Site investigator | Evaluation of participants and/or data management |
| Ruíz Martínez, Javier | Hospital Universitario Donostia, San Sebastián, Spain | Site investigator | Evaluation of participants and/or data management |
| Sánchez Alonso, Pilar | Hospital Universitario Puerta de Hierro, Madrid, Spain | Site investigator | Evaluation of participants and/or data management |
| Sánchez-Carpintero, Macarena | Complejo Hospitalario Universitario de Ferrol (CHUF), Ferrol, A Coruña, Spain | Site investigator | Neuroimaging studies |
| Sánchez Díez, Gema | Hospital Universitario Ramón y Cajal, Madrid, Spain | Site investigator | Evaluation of participants and/or data management |
| Sánchez Rodríguez, Antonio | Hospital Universitario Marqués de Valdecilla, Santander, Spain | Site investigator | Evaluation of participants and/or data management |
| Santacruz, Pilar | Hospital Clínic de Barcelona, Barcelona, Spain | Site investigator | Evaluation of participants and/or data management |
| Santos García, Diego | CHUAC, Complejo Hospitalario Universitario de A Coruña | Coordinator of the Project | Coordination of the COPPADIS-2015 |
| Segundo Rodríguez, José Clemente | Complejo Hospitalario de Toledo, Toledo, Spain | Site investigator | Evaluation of participants and/or data management |
| Seijo, Manuel | Complejo Hospitalario Universitario de Pontevedra (CHOP), Pontevedra, Spain | Site investigator / PI | Coordination at the center  Evaluation of participants and/or data management |
| Sierra, María | Hospital Universitario Marqués de Valdecilla, Santander, Spain | Site investigator | Evaluation of participants and/or data management |
| Solano, Berta | Institut d'Assistència Sanitària (IAS) - Instituí Cátala de la Salud. Girona, Spain | Site investigator / PI | Coordination at the center  Evaluation of participants and/or data management |
| Suárez Castro, Ester | Complejo Hospitalario Universitario de Ferrol (CHUF), Ferrol, A Coruña, Spain | Site investigator | Evaluation of participants and/or data management |
| Tartari, Juan Pablo | Hospital Universitari Mutua de Terrassa, Terrassa, Barcelona, Spain | Site investigator | Evaluation of participants and/or data management |
| Valero, Caridad | Hospital Arnau de Vilanova, Valencia, Spain | Site investigator | Evaluation of participants and/or data management |
| Vargas, Laura | Hospital Universitario Virgen del Rocío, Sevilla, Spain | Site investigator | Evaluation of participants and/or data management |
| Vela, Lydia | Fundación Hospital de Alcorcón, Madrid, Spain | Site investigator / PI | Coordination at the center  Evaluation of participants and/or data management |
| Villanueva, Clara | Hospital Universitario Clínico San Carlos, Madrid, Spain | Site investigator | Evaluation of participants and/or data management |
| Vives, Bárbara | Hospital Universitario Son Espases, Palma de Mallorca, Spain | Site investigator | Evaluation of participants and/or data management |

*Neurology Department, Institute of Neuroscience, Vithas Madrid La Milagrosa University Hospital, Vithas Hospital Group. Madrid, Spain.

**Neurology Department, Hospital Universitario Lucus Augusti, Lugo, Spain.
