# Supplementary material for: Dysphagia in Parkinson´s disease. A 5-year follow-up study
Source: Neurol Sci. 2025 Feb 19;46(6):2637–53. doi: 10.1007/s10072-025-08027-8 (PMC12084275; doi:10.1007/s10072-025-08027-8)
Supplement: Supplementary file 2 — Supplementary file2 (PDF 383 KB) [file 10072_2025_8027_MOESM2_ESM.pdf]

XUNTA DE GALICIA
CONSELLERÍA DE SANIDADE
Secretaría Xeral Técnica

Edificio Administrativo San Lázaro
15703 SANTIAGO DE COMPOSTELA
Teléfono: 881542726 - Fax: 881542728
www.sergas.es

galicia

DICTAMEN DEL COMITÉ AUTONÓMICO DE ÉTICA DE LA INVESTIGACIÓN DE GALICIA

Paula M. López Vázquez, Secretaria del Comité Autonómico de Ética de la Investigación de Galicia

CERTIFICA:

Que este Comité evaluó en su reunión del día 27/11/2014 el estudio:

Título: COPPADIS-2015,COhort of Patients with Parkinson´s DIsease inSpain, 2015
Promotor: Diego Santos Garcia
Tipo de estúdio: EPA-SP
Version: Protocolo COPPADIS.2015 V1 y Documentos de consentimiento informado v2
Código del Promotor: COH-PAK-2014-01
Código de Registro: 2014/534

Y, tomando en consideración las siguientes cuestiones:

- La pertinencia del estudio, teniendo en cuenta el conocimiento disponible, así como los requisitos legales aplicables, y en particular la Ley 14/2007, de investigación biomédica, el Real Decreto 1716/2011, de 18 de noviembre, por el que se establecen los requisitos básicos de autorización y funcionamiento de los biobancos con fines de investigación biomédica y del tratamiento de las muestras biológicas de origen humano, y se regula el funcionamiento y organización del Registro Nacional de Biobancos para investigación biomédica, la ORDEN SAS/3470/2009, de 16 de diciembre, por la que se publican las Directrices sobre estudios Posautorización de Tipo Observacional para medicamentos de uso humano, y la Circular nº 07 / 2004, investigaciones clínicas con productos sanitarios.
- La idoneidad del protocolo en relación con los objetivos del estudio, justificación de los riesgos y molestias previsibles para el sujeto, así como los beneficios esperados.
- Los principios éticos de la Declaración de Helsinki vigente.
- Los Procedimientos Normalizados de Trabajo del CEIC de Galicia

Emite un **INFORME FAVORABLE** para la realización del estudio por el/la investigador/a del centro:

Centros	Investigadores Principales
C.H. Universitario de Ferrol	Diego Santos García
CHU Lucus Augusti	Jessica González
Hospital da Costa	Luis Manuel López Díaz
C.H.U Vigo	Maria Gema Alonso Losada
CHU Pontevedra	Manuel Seijo Martínez

DE ASÍS A COMPOSTELA
800
1214 · 2014

XUNTA DE GALICIA
CONSELLERÍA DE SANIDADE
Secretaría Xeral Técnica

Edificio Administrativo San Lázaro
15703 SANTIAGO DE COMPOSTELA
Teléfono: 881542726 - Fax: 881542728
www.sergas.es

galicia

Y HACE CONSTAR QUE:

1. El CAEIG cumple los requisitos legales vigentes (R.D 223/2004 por el que se regulan los ensayos clínicos con medicamentos, y la Ley 14/2007 de Investigación Biomédica).
2. El CAEIG tanto en su composición como en sus PNTs cumple las Normas de Buena Práctica Clínica (CPMP/ICH/135/95).
3. La composición actual del CAEIG es:

Manuel Portela Romero. (Presidente). Médico Especialista en Medicina Familiar y Comunitaria.
Irene Zarra Ferro. (Vicepresidenta). Farmacéutica de Atención Especializada.
Paula Mª López Vázquez, (Secretaria). Médico Especialista en Farmacología Clínica.
Juan Vázquez Lago (Secretario Suplente). Médico Especialista en Medicina Preventiva y Salud Pública.
Jesús Alberdi Sudupe. Médico especialista en Psiquiatría.
Rosendo Bugarín González. Médico Especialista en Medicina Familiar y Comunitaria.
Juan Casariego Rosón. Médico Especialista en Cardiología.
Xoán X. Casas Rodríguez. Médico Especialista en Medicina Familiar y Comunitaria.
Juana Mª Cruz del Río. Trabajadora Social.
Juan Fernando Cueva Bañuelos. Médico Especialista en Oncología Médica.
José Álvaro Fernández Rial. Médico Especialista en Medicina Interna.
José Luis Fernández Trisac. Médico Especialista en Pediatría.
Mª José Ferreira Díaz. Diplomada Universitaria de Enfermería
Marta Gil Pérez. Licenciada en Derecho. Miembro externo
Pilar Gayoso Diz. Médico Especialista en Medicina Familiar y Comunitaria.
Agustín Pía Morandeira. Farmacéutico de Atención Primaria
Salvador Pita Fernández. Médico Especialista en Medicina Familiar y Comunitaria.
Carmen Rodríguez-Tenreiro Sánchez. Licenciada en Farmacia.
Susana María Romero Yuste. Médico Especialista en Reumatología.
Mª Asunción Verdejo González. Médico Especialista en Farmacología Clínica.

En Santiago de Compostela, a 02 de diciembre de 2014

Paula M. López Vázquez

DE ASIS A COMPOSTELA
800
1214 · 2014
