# Supplementary material for: Dysphagia in Parkinson´s disease. A 5-year follow-up study
Source: Neurol Sci. 2025 Feb 19;46(6):2637–53. doi: 10.1007/s10072-025-08027-8 (PMC12084275; doi:10.1007/s10072-025-08027-8)
Supplement: Supplementary file 3 — Supplementary file3 (PDF 348 KB) [file 10072_2025_8027_MOESM3_ESM.pdf]

COPPADIS-2015, COhort of Patients with PArkinson´s DIsease in Spain, 2015

He sido informado por el profesional de salud abajo mencionado:

- Sobre las ventajas e inconvenientes de este procedimiento.
- Sobre el lugar de obtención, almacenamiento y el proceso que seguirán los datos personales y las muestras.
- Que mis muestras y datos personales serán proporcionados de forma anónima a los investigadores que trabajen con ellas.
- Que en cualquier momento puedo revocar mi consentimiento y solicitar la eliminación de mis datos personales y muestras que permanezcan almacenados en el Biobanco.
- Que en cualquier momento puedo solicitar información genérica sobre los estudios para los que se han utilizado los productos de mis muestras de sangre.
- Que tengo derecho de acceso a mis datos personales archivados en el Biobanco.
- Que tengo derecho a solicitar el resultado del estudio de resonancia magnética (RM) craneal realizado.
- Que tengo derecho a solicitar los resultados de los estudios moleculares séricos realizados con mis muestras.
- Que tengo derecho a solicitar los resultados de los estudios genéticos realizados con mis muestras.
- Que no seré informado de los resultados específicos de esos estudios a excepción de que yo solicite el informe de los resultados genéticos.
- Que he comprendido la información recibida y he podido formular todas las preguntas que he creído oportunas.

  SI □ **NO** □

Acepto participar (ya sea como paciente, cuidador principal o control según proceda) en las siguientes opciones del estudio:

- Evaluación clínica (exploración y/o cuestionarios):
  SI □ **NO** □
- Realización de estudio de neuroimagen (RM craneal) y extracción de sangre para estudios genéticos y moleculares:
  SI □ **NO** □

Firma del paciente:

Nombre del paciente…………………………………………………………………………………………………

Firma:

Fecha:

Firma del representante legal:

Nombre del representante legal……………………………………………………………………………….

Firma:

Fecha:

Firma de la persona que solicita el consentimiento:

Nombre de la persona que solicita el consentimiento…………………………………………………

Firma:

Fecha:
