# Supplementary material for: Dysphagia in Parkinson´s disease. A 5-year follow-up study
Source: Neurol Sci. 2025 Feb 19;46(6):2637–53. doi: 10.1007/s10072-025-08027-8 (PMC12084275; doi:10.1007/s10072-025-08027-8)
Supplement: Supplementary file 4 — Supplementary file4 (PDF 299 KB) [file 10072_2025_8027_MOESM4_ESM.pdf]

COPPADIS-2015, COhort of Patients with PArkinson´s DIsease in Spain, 2015

Título del estudio: COhort of Patients with PArkinson´s DIsease in Spain, 2015.

Instituciones en las que se va a llevar a cabo: en diferentes centros hospitalarios del territorio nacional (España). El requisito imprescindible será que el investigador principal participante en cada centro tenga experiencia y competencias en el diagnóstico y manejo habitual en la práctica clínica diaria de los pacientes con enfermedad de Parkinson.

Investigador responsable: Diego Santos García.

Este documento tiene por objeto ofrecerle información sobre una investigación en la que se le invita a participar y que fue aprobada por el Comité Ético de Investigación Clínica de Galicia. Antes de decidir, debe recibir Vd información personalizada del investigador, leer este documento y hacer todas las preguntas que sean necesarias para comprender los detalles sobre el estudio. Puede llevarse el documento, consultarlo con otras personas, y tomarse el tiempo necesario para decidir si desea participar o no. La participación en este estudio es completamente voluntaria. Si acepta hacerlo, puede cambiar de parecer retirando el consentimiento en cualquier momento sin obligación de dar explicaciones. Su decisión no afectará en modo alguno a la relación con sus médicos ni a la asistencia sanitaria a la que Vd tiene derecho.

1. ¿Cuál es el propósito de este estudio?

El propósito es avanzar en el conocimiento de diferentes aspectos importantes de la enfermedad de Parkinson. El estudio contempla (1) conocer con detalle los síntomas que presenta el paciente con enfermedad de Parkinson en un momento dado y cómo evolucionan los mismos en el tiempo, (2) conocer que repercute en la calidad de vida del paciente y carga del cuidador y provoca que sea peor e (3) intentar identificar marcadores clínicos, de neuroimagen o genéticos que

puedan ser útiles tanto en el diagnóstico como en la estimación de la evolución de la enfermedad.

2. ¿Por qué me ofrecen a mí participar?

Porque Vd cumple con los criterios para poder participar en este estudio (hay unos criterios de inclusión y exclusión que le habrán explicado o le explicarán y no toda persona aunque quisiera puede por lo tanto participar). Su participación será voluntaria. Participando estará contribuyendo a progresar en el conocimiento científico sobre la enfermedad de Parkinson. Igualmente, su participación podría proporcionar información a su médico que le facilite un mejor manejo de su enfermedad. Sin embargo, la no participación voluntaria no afectaría, por supuesto, al trato o manejo habitual por parte de su especialista.

3. ¿En qué consiste mi participación?

Si participa como **paciente**, le serán realizadas una entrevista con cuestiones relacionadas con su enfermedad que incluirá contestar a diferentes cuestionarios y/o escalas validado/as así como una exploración neurológica orientada a conocer el grado de afectación que presenta por el Parkinson. Igualmente serán recogidos datos de su historial clínico presentes en la historia clínica. Si acepta participar además en la realización de estudios complementarios, se le realizará un estudio de resonancia magnética (RM craneal) sin contraste y extracción de 30 mL de sangre para estudios genéticos y análisis de diferentes marcadores moleculares; 6 mL se utilizarán para analizar marcadores moleculares en sangre, siendo las muestras enviadas a un laboratorio común en Barcelona (REFERENCE LABORATORY, www.reference-laboratory.es); los otros 24 mL se congelarán y se almacenarán en un Biobanco de muestras biológicas (Biobanco del Hospital Universitario Virgen del Rocío del Sistema Público de Andalucía). De los 24 mL, 20 mL serán utilizados para estudios genéticos mientras que los otros 4 mL se contemplan para analizar futuros biomarcadores no contemplados en el proyecto

actual. Podría participar sólo en la parte clínica o en las dos según su voluntad y criterios del estudio.

Si participa como **cuidador principal**, su participación se limitará a una entrevista que incluirá preguntas sencillas así como responder a algunos cuestionarios y/o escalas validado/as.

Si participa como **control**, le serán realizadas una entrevista que incluirá contestar a diferentes cuestionarios y/o escalas validado/as. Si acepta participar además en la realización de estudios complementarios, se le realizará un estudio de RM craneal sin contraste y extracción de 30 mL de sangre para estudios genéticos y análisis de diferentes marcadores moleculares; 6 mL se utilizarán para analizar marcadores moleculares en sangre, siendo las muestras enviadas a un laboratorio común en Barcelona (REFERENCE LABORATORY, www.reference-laboratory.es); los otros 24 mL se congelarán y se almacenarán en un Biobanco de muestras biológicas (Biobanco del Hospital Universitario Virgen del Rocío del Sistema Público de Andalucía). De los 24 mL, 20 mL serán utilizados para estudios genéticos mientras que los otros 4 mL se contemplan para analizar futuros biomarcadores no contemplados en el proyecto actual. Podría participar sólo en la parte clínica o en las dos según su voluntad y criterios del estudio.

Toda la información recogida a lo largo de la presente investigación (clínica, neuroimagen, molecular y genética), será archivada en una base de datos especial dedicada únicamente a esta investigación y podrá ser incorporada en su historia clínica rutinaria. Este proyecto tendrá una duración total estimada de 5 años. Los investigadores podrán decidir finalizar el estudio antes de lo previsto o bien interrumpir su participación en el mismo por aparición de nueva información relevante, por motivos de seguridad, o por incumplimiento de los procedimientos de estudio.

La duración del estudio son 5 años. Se realizará una evaluación anual que incluirá exploración (pacientes) y cuestionarios y/o escalas (pacientes, cuidadores y controles). Al final de los 5 años si realizó al inicio los estudios complementarios (RM craneal y extracción de sangre), volverá a repetir los mismos (RM craneal y

extracción de sangre para análisis de marcadores moleculares). Como ya se ha comentado, la participación es voluntaria y podrá abandonar el estudio si lo desea en cualquier momento sin dar explicaciones y sin que ésto le repercuta en cuanto a la atención y trato de su médico.

4. ¿Qué riesgos o incovenientes tiene participar?

Es un estudio observacional en el cual Vd como sujeto participante no será sometido a ningún tratamiento aprobado ni en desarrollo para conseguir una indicación determinada. Por lo tanto, los riesgos derivados del intervencionismo no existen en lo que a tratamiento se refiere dado que este punto no lo contempla el estudio. Por supuesto, puede haber complicaciones secundarias a algún medicamento que su médico le prescriba durante el seguimiento que aunque en realidad formaría parte de la actividad asistencial rutinaria se recogería en el estudio.

La exploración neurológica incluyendo la evaluación motora de la enfermedad de Parkinson es la habitual o similar a la que se suele realizar en una consulta de Neurología y/o específica de trastornos del movimiento. En el caso de tener que acudir sin medicación desde el día anterior para ser evaluado en situación OFF medicación y ON medicación, como ya sabrá su situación motora por la mañana probablemente le ocasione mayor dependencia y limitaciones, pudiendo requerir por lo tanto de alguien para poder acudir a la consulta (cuando en otras condiciones a lo mejor no). Esto se lo deberán explicar claramente antes de participar para que lo entienda y asuma potenciales complicaciones de tal situación (dolor, limitación para caminar con riesgo de caídas, etc.). Sin embargo, este tipo de evaluaciones (sin y con medicación) son también habituales en la práctica clínica y se realizan por ejemplo en pacientes que se plantean operar del Parkinson. Es en realidad la mejor forma para que su médico conozca su estado y como la enfermedad le afecta, dado que una visita puntual en un momento dado con la medicación tomada previamente no representa la realidad (24 horas del enfermo de Parkinson). Con respecto a la exploración no motora, uno de los

inconvenientes que pueda haber es el cansancio y estrés en relación con el interrogatorio y duración de la evaluación. El protocolo contempla fraccionar en dos la evaluación si fuera preciso. Por otra parte, la información sobre diferentes aspectos (memoria, estado de ánimo, sueño, conducta, calidad de vida, etc.) que obtendrá el médico le ayudarán de nuevo a conocer mucho mejor su estado en relación con la enfermedad de Parkinson (comparado con la información que se suele obtener en una consulta rutinaria asistencial).

La extracción de sangre (para analizar marcadores moleculares y almacenar muestras que en el futuro se utilizarán para realizar estudios genéticos y/o marcadores moleculares) puede conllevar ligeras molestias derivadas del pinchazo o la aparición de un pequeño hematoma. Puede ser que, como en cualquier otra extracción de sangre, pueda sufrir ligeras molestias transitorias en el lugar de punción o algún mareo pasajero.

El estudio de neuroimagen es una RM (resonancia magnética) craneal sin contraste. No es una prueba que emita radiaciones (y perjudicial en este sentido, como puede ser la realización de un scanner). Tampoco se administrará contraste, que en ocasiones puede generar alguna complicación. El mayor inconveniente será el tener que estar en reposo tumbado en la camilla de la RM durante un tiempo aproximado de 20 minutos sin moverse. Esto en ocasiones puede generar angustia o estrés y obliga a parar el procedimiento (más habitual en personas con ansiedad o claustrofobia).

La participación en el estudio implica un riesgo de impacto psicológico adverso, ansiedad o depresión en relación posible con los resultados. En relación con los resultados de los estudios genéticos, marcadores moleculares en sangre y RM craneal, no se le proporcionará dicha información. Sólo el investigador principal del estudio conocerá esa información. Sin embargo, tiene derecho a solicitar información sobre los resultados de los mismos si lo deseara. El estudio contempla un seguro de responsabilidad civil para aquellos participantes que realicen los estudios complementarios (RM craneal y extracción de sangre) con intención de cubrir posibles complicaciones derivadas de las mismas.

Existe un riesgo de confidencialidad. La información clínica y genética obtenida en este proyecto será almacenada en una base de datos protegida por la legislación vigente, custodiada bajo la responsabilidad de los investigadores e instituciones responsables. Los resultados de esta investigación se podrán difundir en revistas, bases de datos médicas y foros científicos. Aunque no se desvelarán datos personales que pudieran reconocerle, puede existir cierto riesgo de que, de manera indirecta, alguien pudiera llegar a reconocer su identidad.

5. ¿Obtendré algún beneficio por participar?

Este estudio pretende avanzar en el conocimiento de aspectos clave de la enfermedad de Parkinson como el diagnóstico o la progresión natural de la enfermedad. Es posible que estos descubrimientos puedan suponer un beneficio en el manejo de la enfermedad para Vd mismo u otros pacientes con Parkinson en el futuro. No se contempla un beneficio de tipo económico por participar. El manejo por parte de su especialista no cambiará por el hecho de participar o no en el estudio.

6. ¿Recibiré la información que se obtenga del estudio?

Si Vd. lo desea, se le facilitará un resumen de los resultados del estudio. Puesto que estos datos pueden tener implicaciones que afecten al futuro de su salud o la de sus familiares, y además algunos resultados pueden no tener una interpretación clara, si desea disponer de ellos, le serán proporcionados directamente por los médicos de la investigación junto con un adecuado asesoramiento.

7. ¿Se publicarán los resultados de este estudio?

Los resultados de este estudio serán remitidos a publicaciones científicas para su difusión. Asimismo, algunos datos clínicos o genéticos podrán difundirse

entre la comunidad científica a través de bases de datos en internet, sin asociar ningún dato personal que pueda llevar a la identificación de los pacientes.

8. ¿Cómo se protegerá la confidencialidad de mis datos?

Toda la información que se recoja de su historia clínica se mantendrá de manera estrictamente confidencial y no se divulgará ningún dato sin su consentimiento. Si bien se mantendrá su privacidad, algunas partes relevantes de su historia clínica podrían ser revisadas por personal autorizado del hospital o por las autoridades sanitarias. Los datos obtenidos serán analizados por parte de un comité de Neurólogos expertos y también puede ser utilizado para presentar informes a las autoridades sanitarias. Estos análisis también podrían publicarse en revistas médicas. Su nombre no será divulgado ni mencionado en ninguna publicación que se origine como resultado de este estudio.

El tratamiento, comunicación y cesión de sus datos se hará conforme a lo dispuesto por la Ley Orgánica 15/1999, del 13 de diciembre, de protección de datos de carácter personal. Como participante, Vd podrá ejercer su derecho de acceso, rectificación, cancelación y oposición contactando con el investigador principal de su centro. Sólo el equipo investigador y sus colaboradores así como las autoridades sanitarias, que tienen deber de garantizar la confidencialidad, tendrán acceso a todos los datos recogidos por el estudio. En el caso de que alguna información sea transmitida a otros países, se realizará con un nivel de protección de los datos equivalente, como mínimo, al exigido por la normativa de nuestro país.

9. ¿Qué ocurrirá con las muestras obtenidas?

Sus muestras serán guardadas en los laboratorios del Biobanco del Hospital Universitario Virgen del Rocío de Sevilla (Biobanco del Sistema Sanitario Público de Andalucía). Se almacenarán de forma codificada, lo que quiere decir que las muestras poseen un código que permite identificar al donante. Esta información está a cargo de los investigadores principales y sólo podrán acceder a ellas los

miembros del equipo investigador, sus colaboradores y las autoridades sanitarias en el ejercicio de sus funciones. Para más información referente a este punto puede consultar el documento “FORMULARIO DE INFORMACIÓN Y CONSENTIMIENTO INFORMADO ESCRITO” del “Biobanco del Sistema Sanitario Público de Andalucía”.

10. ¿Existen intereses económicos en este estudio?

Vd. no será retribuido por participar. En el caso de que de los resultados del estudio se derivasen productos comerciales o patentes, Vd no participará de los beneficios económicos originados. Los investigadores del estudio recibirán una compensación económica por su actividad.

11. ¿Quién me puede dar más información?

Para más información puede contactar con el investigador principal de su centro correspondiente. En caso de creerlo oportuno puede contactar con el promotor e investigador principal y coordinador del estudio, Diego Santos García (diegosangar@yahoo.es; 646-173341).
